# A patient with lissencephaly, developmental delay, and infantile spasms, due to de novo heterozygous mutation of *KIF2A*

**DOI:** 10.1002/mgg3.236

**Published:** 2016-09-28

**Authors:** Guoling Tian, Ana G. Cristancho, Holly A. Dubbs, Grant T. Liu, Nicholas J. Cowan, Ethan M. Goldberg

**Affiliations:** ^1^ Department of Molecular Pharmacology and BiochemistryNew York University Langone Medical centerNew YorkNew York10016; ^2^ Department of PediatricsDivision of NeurologyThe Children's Hospital of PhiladelphiaPhiladelphiaPennsylvania19104; ^3^ Neuro‐Opthalmology ServiceDivision of OphthalmologyThe Children's Hospital of PhiladelphiaPhiladelphiaPennsylvania19104; ^4^ Department of NeurologyThe Perelman School of Medicine at The University of PennsylvaniaPhiladelphiaPennsylvania19104; ^5^ Department of NeuroscienceThe Perelman School of Medicine at The University of PennsylvaniaPhiladelphiaPennsylvania19104

**Keywords:** Brain development, *KIF2A*, lissencephaly, microtubules

## Abstract

**Background:**

Microtubules are dynamic polymers of *α*/*β* tubulin heterodimers that play a critical role in cerebral cortical development, by regulating neuronal migration, differentiation, and morphogenesis. Mutations in genes that encode either *α*‐ or *β*‐tubulin or a spectrum of proteins involved in the regulation of microtubule dynamics lead to clinically devastating malformations of cortical development, including lissencephaly.

**Methods:**

This is a single case report or a patient with lissencephaly, developmental delay, nystagmus, persistent hyperplastic primary vitreous, and infantile spasms, and undertook a neurogenetic workup. We include studies of mutant function in *Escherichia coli* and HeLa cells.

**Results:**

The patient was found to have a novel de novo mutation in kinesin family member 2A (*KIF2A*). This mutation results in a substitution of isoleucine at a highly conserved threonine residue within the ATP‐binding domain. The KIF2A p.Thr320Ile mutant protein exhibited abnormal solubility, and KIF2A p.Thr320Ile overexpression in cultured cells led to the formation of aberrant microtubule networks.

**Conclusion:**

Findings support the pathogenic link between *KIF2A* mutation and lissencephaly, and expand the range of presentation to include infantile spasms and congenital anomalies.

## Introduction

Lissencephaly refers to the almost complete absence of normal gyration and sulcation of the cerebral cortex, and is frequently associated with microcephaly, severe to profound developmental delay/intellectual disability, and medically intractable epilepsy. A more complete knowledge of the genetic etiology of lissencephaly is critical toward furthering the understanding of pathways governing normal cortical development and the mechanisms that underlie malformations of cortical development, and is important clinically for the purposes of providing the best possible prognostic information and recurrence risk estimates to patients and their families.

The advent of massively parallel/next‐generation sequencing (NGS) technology has driven a recent surge in the discovery of genes causally associated with brain malformations, and there are now over 20 genes associated with lissencephaly (reviewed in Liu 2011; Fry et al. 2014). Implicated genes are involved in a variety of processes, including neuronal and glial proliferation and apoptosis, neuronal migration, and/or postmigrational development (Poretti et al. 2014). Many of these genes exert such effects through regulation of cytoskeletal dynamics via microtubule‐driven events that are required for neuronal proliferation, axonal guidance and dendritic sprouting (Liu 2011; Moon and Wynshaw‐Boris 2013). This specifically includes the tubulin (*TUBA1A*,* TUBB2B*,* TUBB3*, and *TUBG1*) and kinesin (*KIF2A*,* KIF5C*) genes (Poirier et al. 2013). Here we provide the second report and third documented case of lissencephaly due to a novel mutation in the *KIF2A* gene (OMIM #602591).

## Materials and Methods

### Human subjects

This is a single case report. Appropriate informed consent was obtained from the patient's legal guardians.

### Expression studies in *Escherichia coli*

The ATP‐binding domains (amino acids 153‐553, PDB 2GRY) of C‐terminally His_6_‐tagged wild type and KIF2A p.Thr320Ile (T320I) were expressed in *E. coli* BL21DE3 host cells. Soluble extracts were prepared from equal masses of bacteria as described previously (Poirier et al. 2013), and the recombinant protein purified by selection on and elution from affinity columns (TALON Co.). The relative abundance of the recombinant proteins in the samples from soluble and insoluble fractions was assessed by fractionation on 8.5% SDS‐PAGE.

### Expression of in HeLa cells

Plasmids engineering for the CMV‐driven expression of full‐length wild type (Origene, Inc., Rockville, MD, U.S.A., SC117315) and mutant *KIF2A* c.959C>T were used to transfect HeLa cells. Reference sequence for *KIF2A* (GenBank NM_004520.4) is according to the Human Genome Variation Society (http://varnomen.hgvs.org).

## Results

### Clinical course and diagnostic workup of patient

**Figure 1:**
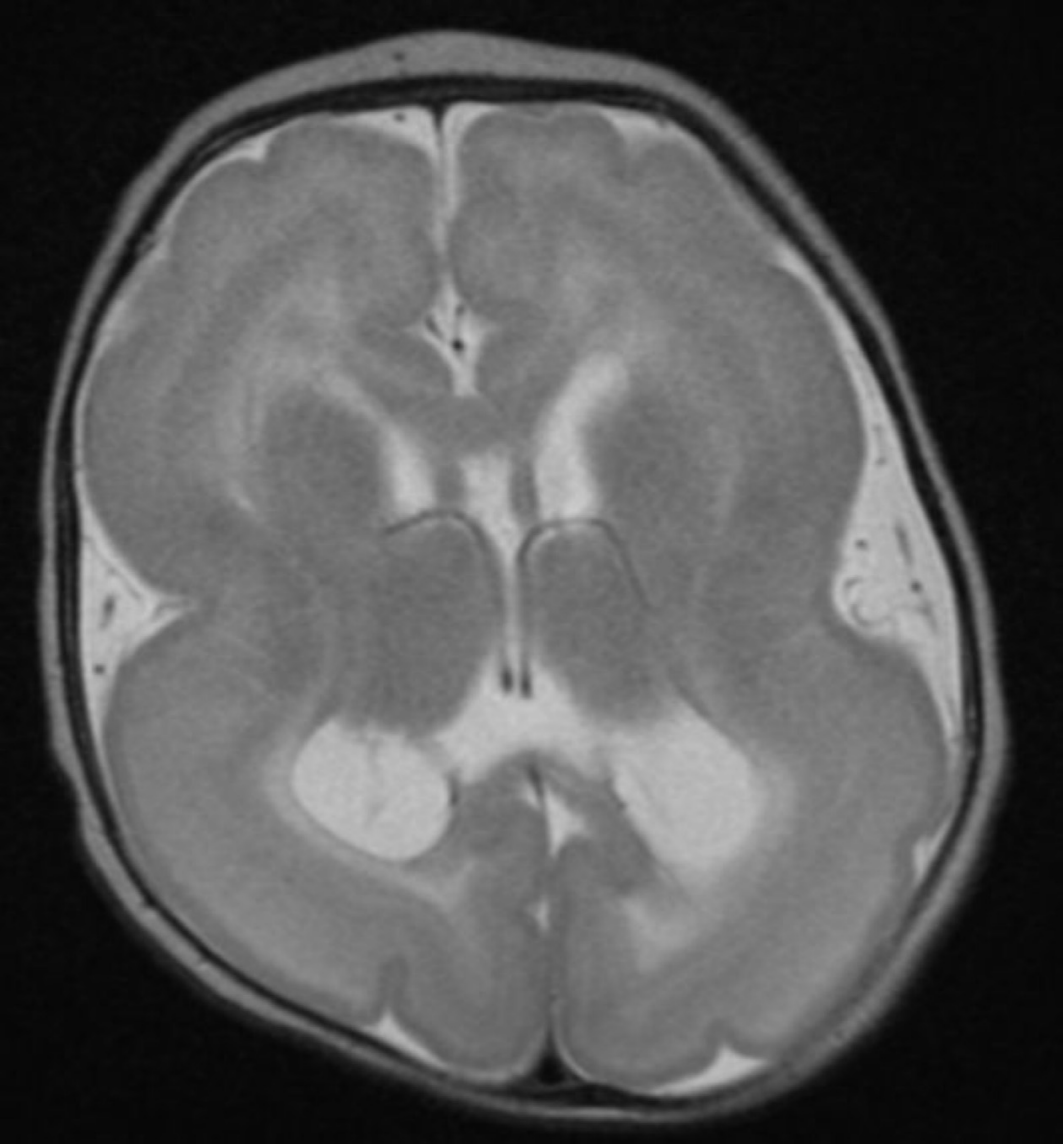
MRI of the brain reveals lissencephaly. Shown is a selected image from an axial T2‐weighted MRI scan of the brain performed at 2 months 1 week of age. There is a paucity of cortical sulcation with broad, thickened gyri, and a near‐smooth and thickened appearance of the surface of the cerebral cortex, perhaps with a slight posterior predominance. The brain exhibits the typical “hourglass” configuration of lissencephaly. T2‐weighted scans further reveal a hyperintense, cell‐sparse zone within the cerebral cortex further characteristic of lissencephaly. The corpus callosum is dysplastic.

The patient is an ex‐37 week gestation female infant born to nonconsanguinous parents without complications. Birth history was remarkable for oligohydramnios, with elective cesarean section for breech presentation. At 2 months of age, the patient was noted to exhibit abnormal eye movements, and was found to have downgaze and horizontal nystagmus, ptosis, unilateral microphthalmia, and persistent hyperplastic primary vitreous (PHPV). MRI of the brain revealed lissencephaly, perhaps with a slight posterior predominance, with a dysplastic corpus callosum, and slightly diminutive brainstem (Fig. 1); MRI of the orbits (not shown) demonstrated bilateral PHPV with decreased size of the left globe and thin optic nerves bilaterally. Neurological examination was further significant for relative microcephaly (with a head circumference of 35.8 cm at age 2 months, at approximately the 1st percentile for age) and central hypotonia. A serum creatine kinase (CK) level was normal at 76 units/L. At 8 months of age, the patient was noted to have clusters of extensor spasms, most typically at sleep/wake transitions. These were confirmed to be infantile spasms, with associated electrodecrements, and periods of intermittent hypsarrhythmia noted on continuous video electroencephalogram (EEG; not shown). The patient was initially placed on levetiracetam, then topiramate, without effect; valproic acid was then initiated with subsequent addition of clobazam, which had a partial, but incomplete, effect. Vigabatrin produced near‐cessation of clinical spasms.

The patient's clinical status was also remarkable for profound developmental delay. At age 9 months, the patient was non‐verbal, and did not coo or babble; she was visually impaired, with roving eye movements and intermittent fine horizontal and vertical nystagmus. There was significant central hypotonia with poor head control, and she did not reach for objects of interest and did not roll over.

### Clinical genetic results

Genetic testing included a commercial next‐generation (NextGen) sequencing panel assessing known causes of brain malformations, which revealed a heterozygous c.959C>T (p.Thr320Ile) missense mutation in *KIF2A*, a member of the kinesin family. This was subsequently shown to be de novo. This mutation is not present in over 60,000 control subjects in the Exome Aggregation Consortium (ExAC; Broad Institute, Cambridge, MA). This mutation is close to but distinct from those reported in the only two previously described patients with *KIF2A* mutation and lissencephaly (Poirier et al. 2013). The point mutation leads to transition from a polar, uncharged threonine residue to a hydrophobic isoleucine residue, located in the ATP‐binding domain of the KIF2A protein. This residue exhibits a high degree of evolutionary conservation. Furthermore, the mutation is predicted to be damaging to protein structure based on in silico algorithms, including PolyPhen‐2 (with a score of 0.986).

### Expression studies of mutant protein in heterologous systems

**Figure 2:**
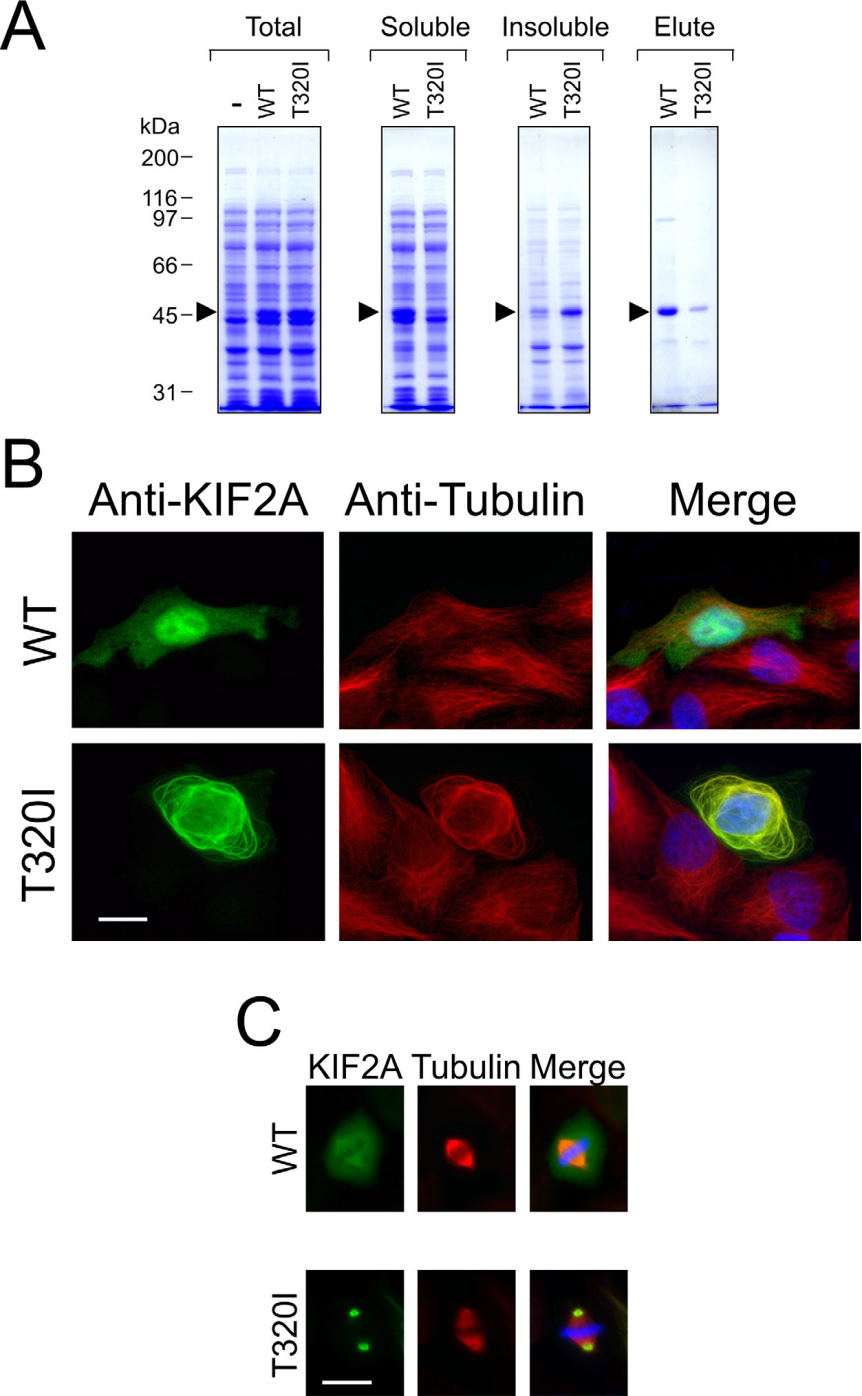
Abnormal function of mutant KIF2A. (A) Reduced solubility of the mutant KIF2A p.Thr320Ile ATP‐binding domain. The arrow denotes the migration position of the recombinant protein. Note the approximately equal expression of wild‐type and mutant protein in whole extracts of host cells (“Total”); the increased relative abundance of the wild‐type protein in the soluble fraction; the increased abundance of the mutant protein in the insoluble fraction; and the substantially reduced yield of purified T320I mutant protein (“Elute”) compared to the wild‐type control. Molecular size markers are shown *at left*. (B, C) Expression of wild‐type and mutant KIF2A in interphase and mitotic HeLa cells. Expression of wild‐type mutant *KIF2A* c.959C>T transgenes was examined by immunofluorescence microscopy using an anti‐KIF2A antibody (shown in green; left panels in B and C) together with an anti‐*α*‐tubulin antibody to detect the microtubule network (shown in red; middle panels in B and C). DNA (shown in blue in the right panels in B and C) was visualized by staining with Hoechst. Note that in interphase cells (B), wild‐type KIF2A is present as a diffuse nuclear and cytosolic stain, while KIF2A p.Thr320Ile colocalizes with the microtubule network, often resulting in morphologically abnormal microtubules that are unusually thick and that tend to have a circumferential organization. In mitotic cells (C), the wild‐type protein exhibits a diffuse stain superimposed on weakly staining spindle microtubules, while the mutant protein is uniquely localized at the spindle poles. Scale bar in B and C, 20 microns.

KIF2A has been described as a modulator of microtubule stability; depending on phosphorylation state, the protein is capable of either stabilizing microtubules or promoting microtubule depolymerization (Ogawa and Hirokawa 2015). *Kif2a* knockout mice demonstrated abnormal axonal branching with aberrant microtubule function (Homma et al. 2003). To determine whether the *KIF2A* mutation c.959C>T (p.Thr320Ile) leads to protein dysfunction, we first expressed a His_6_‐tagged head domain of wild type and mutant in *E. coli*. We found that the *KIF2A* mutant c.959C>T (p.Thr320Ile) head domain was relatively insoluble compared to the corresponding wild‐type control (Fig. 2A). Full‐length constructs encoding wild‐type KIF2A and mutant *KIF2A* c.959C>T (p.Thr320Ile) were then heterologously expressed by transfection in HeLa cells, which were examined my immunofluorescence to determine the subcellular localization of the transgene. Wild‐type KIF2A exhibited a diffuse nuclear and cytosolic stain without conspicuous microtubule colocalization, consistent with previous reports (Poirier et al. 2013). In contrast, mutant *KIF2A* c.959C>T (p.Thr320Ile) led to a markedly different pattern of cellular localization, with pronounced staining restricted to microtubules; these were abnormally thick and exhibited an aberrant, circumferential organization (Fig. 2B). In addition, in mitotic cells, mutant *KIF2A* c.959C>T (p.Thr320Ile) uniquely localized to the spindle poles, rather than the typical pattern (Ganem and Compton 2004) characterized by diffuse staining superimposed on weakly stained spindles (Fig. 2C). These data support the theory that missense mutations in the ATP‐binding domain of *KIF2A* lead to improper protein folding and/or binding and hydrolysis of ATP with a resulting decrease in available functional protein, possibly by structural destabilization. KIF2A has previously been described as a regulator of central spindle assembly during mitosis, with knockdown leading to asymmetric cell division (Ganem and Compton 2004; Uehara et al. 2013). Such data indicate that KIF2A may be required for modulating both general microtubule organization in the developing brain and appropriate cell division during early neuronal development.

## Discussion

In conclusion, we describe the third patient to date with lissencephaly due to heterozygous de novo mutation in *KIF2A*. The patient presented with microcephaly, profound global developmental delay, nystagmus, and hypotonia, similar to a previous report (Poirier et al. 2013). Our patient additionally had microphthalmia and PHPV, which were not previously described, and may represent a slight expansion of the phenotypic spectrum of this disorder. Functional analysis of mutant protein demonstrated abnormal localization of KIF2A and abnormal microtubule structure.

PHPV is a rare developmental abnormality of the eye and is typically unilateral (Shastry 2009). Unusual cases of bilateral PHPV, as well as microphthalmia, have been previously reported in patients with Walker–Warburg Syndrome (WWS), a severe congenital muscular dystrophy characterized by eye abnormalities as well as brain malformations typically including the so‐called “cobblestone” lissencephaly (Gerding et al. 1993). WWS is genetically heterogenous and is classified as a dystroglycanopathy with the underlying pathological basis being abnormal glycosylation of *α*‐dystroglycan (Godfrey et al. 2011). The patient reported here did not have muscular dystrophy. Expression levels of KIF2A in the eye are not known so far as we are aware, and the relationship between this patient and other patients with WWS and similar eye abnormalities is not clear.

We suspect that additional patients with lissencephaly due to *KIF2A* mutation will be identified with the growing use of targeted clinical diagnostic NextGen sequencing panels for brain malformations and malformations of cortical development.

## Conflict of Interest

None declared.
